# Intense Synaptic Activity Enhances Temporal Resolution in Spinal Motoneurons

**DOI:** 10.1371/journal.pone.0003218

**Published:** 2008-09-16

**Authors:** Rune W. Berg, Susanne Ditlevsen, Jørn Hounsgaard

**Affiliations:** 1 Department of Neuroscience and Pharmacology, University of Copenhagen, Copenhagen, Denmark; 2 Department of Mathematical Sciences, University of Copenhagen, Copenhagen, Denmark; Emory University, United States of America

## Abstract

In neurons, spike timing is determined by integration of synaptic potentials in delicate concert with intrinsic properties. Although the integration time is functionally crucial, it remains elusive during network activity. While mechanisms of rapid processing are well documented in sensory systems, agility in motor systems has received little attention. Here we analyze how intense synaptic activity affects integration time in spinal motoneurons during functional motor activity and report a 10-fold decrease. As a result, action potentials can only be predicted from the membrane potential within 10 ms of their occurrence and detected for less than 10 ms after their occurrence. Being shorter than the average inter-spike interval, the AHP has little effect on integration time and spike timing, which instead is entirely determined by fluctuations in membrane potential caused by the barrage of inhibitory and excitatory synaptic activity. By shortening the effective integration time, this intense synaptic input may serve to facilitate the generation of rapid changes in movements.

## Introduction

Spike timing in nerve cells is determined by temporal integration of synaptic potentials and intrinsic response properties. However, little is known about the timescale of this integration during functional network activity and how it is affected by synaptic events. In the absence of synaptic input the spike afterhyperpolarization (AHP) determines spike timing during repetitive firing. In motoneurons (MNs), the frequency range of this firing is well suited for force regulation in the muscle fibers they innervate [1]. This suggests that main role of AHP is temporal filtering that converts the continuous asynchronous synaptic bombardment to a regular output discharge of action potentials. Furthermore, firing maintained by AHP and other slow intrinsic properties is also appealing because it is a metabolically inexpensive way of shaping the spike patterns to suit particular functions, e.g. spinal motor rhythms [2]–[5].

On the other hand, the AHP and other slow intrinsic properties would impede rapidly changing motor responses and it is not known how resilient they are to a noisy background of synaptic activity. Recent evidence suggests that intrinsic response properties may be shunted by synaptic conductance in cortical and sub-cortical networks [6]–[10]. In the spinal cord of the adult turtle, scratch motor network activity is associated with a dramatic rise in conductance and in fluctuations of the membrane potential (V_m_) in both MNs and interneurons during spiking [11], [12]. This is due to a concurrent intense inhibitory and excitatory synaptic activity [13]. Under these conditions of high synaptic conductance, the temporal resolution is predicted to be enhanced [14]–[16] and the role of slow intrinsic properties becomes less obvious. Surprisingly few experimental studies have explored this interplay between high synaptic conductance, AHP and temporal integration in active networks.

For this reason, we have conducted experiments on spinal motoneurons embedded in a functionally active network during fictive motor behavior. In earlier studies the conductance increase in motoneurons during fictive locomotor and scratch network activity was first measured in vivo in the cat [17], [18] and in the turtle [12]. The isolated spinal cord-carapace preparation from the turtle [19] offers uniquely stable recording conditions in which intrinsic and synaptic conductance changes during network activity can be measured against a background of very low leak conductance [11], [13], [20]. This allows us for the first time to quantify the relative importance of active and passive intrinsic properties and the dynamics of synaptic input for spike timing during functional network activity.

**Figure 1:**
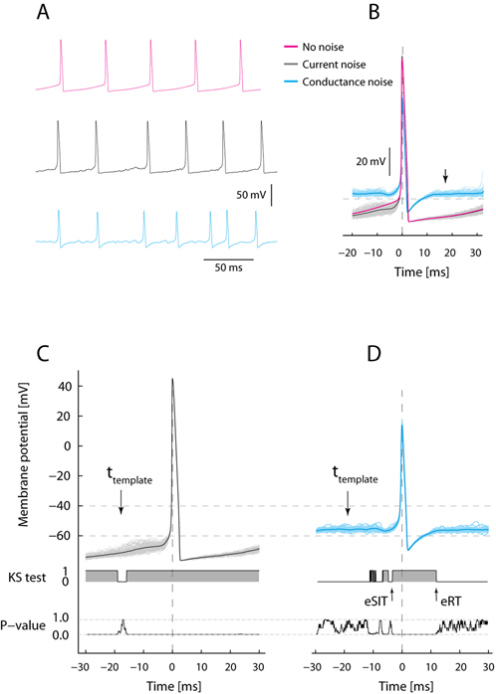
Computer simulation to illustrate firing pattern and AHP in current-based and conductance based models of synaptic input during repetitive firing. (A). Red top trace: Model with constant current injection, no other input. Black middle trace: Model with added current noise with same mean current as in top trace. Blue bottom trace: Model with same mean injected constant current and noise stemming from fluctuating conductance. (B). Spike triggered superimposed spikes. Red, black (n = 243) and blue (n = 72) traces are averages of spikes from (A). Notice the current-noise average closely overlap the no-noise trace (red) whereas the conductance-noise average (blue) rapidly reach pre-spike V_m_-level (see arrow), because of increase in total conductance. (C) and (D) illustrate the statistical quantification of the evolution of V_m_ before and after the spike for traces in (B). A template distribution of V_m_ traces is chosen at an arbitrary time prior to the spike (see arrow at t_template_) for which the distribution at the rest of the time points is compared with. The outcome of comparison is shown as the KS-test trace below, 1 represents acceptance and 0 represents rejection of the hypothesis that they are different. Below is shown the P-values for the KS test. The distribution of V_m_ in (C) is different everywhere, whereas the distribution in (D) is only different up to and immediately after the spike. The time it takes to regain the pre-spike distribution following the spike is referred to as *effective recovery time* (eRT, arrow), while the endmost time of same distribution before the spike is referred to as *effective synaptic integration time* (eSIT, arrow).

We measured the effective integration time in MNs during network activity using a novel statistical approach that quantified the V_m_-fluctuations before and after the action potential. Three temporal features were characterized: the membrane time-constant, *effective synaptic integration time* (eSIT) and the *effective recovery time* (eRT). We define the eSIT as the time it takes to sum up enough synaptic input to cause a spike. The eRT is defined as the time it takes for the V_m_-distribution following a spike to return to the pre-spike condition, i.e. how long it takes the cell to “forget” that a spike has occurred. We report eRT as short as 4 ms during network activity, which is more than a 10-fold decrease compared with quiescent network. Our results show that even prominent intrinsic response properties like the AHP are severely attenuated concurrent with increase in synaptic conductance. For this reason, the contribution of synaptic activity and active membrane properties to network dynamics can only be captured by a conductance-based model [8], [16], [21], [22] (Figure 1). We conclude that a shortening of the recovery time and integration time of motoneurons cause an increase in the temporal resolution of the motor system during activity, which we suggest as a mechanism to facilitate rapidly changing movements.

## Results

The question we ask is how the surge in conductance from intense synaptic bombardment during network activity modifies intrinsic properties exemplified by the spike AHP and shrinks response time and integration time of the neuron. The sensitivity of spike generation and spike pattern to conductance is illustrated in simulations in a simple model (Figure 1). In the absence of synaptic input the regular firing during maintained depolarization in the model neuron was determined by the AHP (red trace in Figure 1A). Synaptic input simulated as current noise, i.e. linear summation of synaptic potentials, resulted in greater variability in spike timing, but did not change the averaged AHP (black trace Figure 1A, B). Thus, current-based synaptic input fluctuations changed neither total conductance nor neuronal integration time. However, when synaptic activity was modeled more realistically as a noisy conductance increase, spike timing was strongly influenced by the stochastic fluctuations in V_m_ (blue trace, Figure 1A, B), the AHP was severely attenuated (arrow in Figure 1B) and the integration time was shortened [22], [23]. Thus, the simple simulation showed that a fluctuating current is not likely to affect the integration time or reduce the importance of the AHP whereas intense and fluctuating synaptic conductance is.

For the purpose of making these statements applicable to quantitative evaluation of experimental data, we developed statistics to capture the temporal features of V_m_ before and after the spike. We define two measures linked to the superposition of spikes (Figure 1C and D). First, the *effective synaptic integration time* (eSIT), defined as the time period prior to spikes during which fluctuations in membrane potential showed a significant depolarizing trend compared with a baseline template (Figure 1D). Likewise, we define the *effective recovery time* (eRT), as the time it takes to regain pre-spike V_m_-levels. Notice that both eRT and eSIT are absent in current noise model whereas they are present in the conductance noise model (cf. Figure 1 C and D).

**Figure 2:**
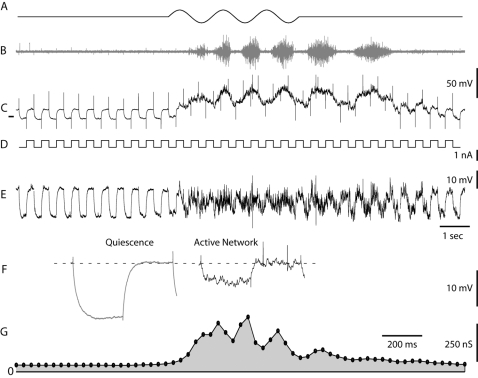
Increase in synaptic conductance during a single scratch epoch. (A) Cutaneous stimulation via sinusoidal movements of a glass rod on the hind-limb pocket skin. (B) Electro-neurogram from hip flexor nerve. (C) V_m_ from intracellular recording. Tick mark indicates V_m_ of −100 mV. (D) Current pulses of −0.7 nA from constant current level of −1.0 nA. (E) High-pass filtered V_m_ from (C) (cut off is 2 Hz). Transient artifacts removed. (F) left, average voltage deflections from (E) during quiescence (n = 14), right during network activity (n = 14). The average increase in conductance is 340%. Notice the occurrence of two spikes. (G) The membrane conductance as a function of time. The peak conductance during network activity is >800% of the conductance during quiescence.

To evaluate whether the neuronal integration time and AHP are substantially affected during intense network activity we measured eSIT and eRT in MN during scratching. The data set consisted of more than 10.000 spikes in 185 scratch epochs in 17 MNs. The analysis is organized as follows. First, we estimated the conductance increase in a sub-sample of MN to confirm that strong synaptic components were present (Figure 2). Next, we applied our statistics to measure the eSIT and eRT and estimated the effective membrane time-constant from V_m_-fluctuations at different levels of synaptic activity. Since intensity of synaptic activity may vary among cells, we compared the eRT with indicators of input, i.e. the effective membrane time constant as well as the smallest inter-spike interval (ISI) for each MN. Finally, since the functional expression of AHP accumulation in motoneurons is spike frequency adaptation (SFA) [4], [24], we also tested for adaptation during each cycle of the scratch epochs.

### Synaptic conductance

A scratch epoch was induced by rhythmic cutaneous stimulation of the skin in the hind-limb pocket and the concurrent synaptic conductance (Figure 2A, B) was estimated from the voltage deflections to injected current pulses (Figure 2C–F) for a subset of MN. Conductance due to action potentials is a potential source of error in these measurements [25]. We therefore avoided spikes by injecting a steady hyperpolarizing current (typically −2 nA) or selected a part of the scratch episode without spikes. The pair of spikes in Figure 2F was left in for illustration, but the sweep was not included in the final estimate. Even with this conservative approach, the estimated conductance increased 2–5 times during scratching (table 1 and Figure 2G)[9]. We attribute this increase to balanced inhibitory and excitatory synaptic input [13] and conclude that temporal processing in MNs could be affected by synaptic conductance during network activity.

**Table 1:** Total input conductance for 5 MN during both quiescence and active network.

Motoneuron number	G_quiescence_ [nS]	G_active_ [nS]	Fraction [100%]
2	73	320	438
3	45	181	405
10	71	224	314
13	20	40	200
14	55	258	470

### Effective synaptic integration time

**Figure 3:**
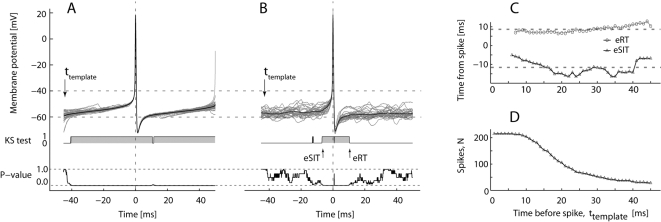
Effective recovery time and synaptic integration time during scratching. Peri-spike V_m_ distribution in quiescence (A) (n = 50) and during scratching (B) (n = 29). Top, superimposed data traces (gray) with mean V_m_ in black. Middle, KS-test. Bottom, P-values for the KS test. The *effective synaptic integration time* (eSIT) and *effective recovery time* (eRT), defined as the time periods before and after the spike, in which the V_m_ distribution is significantly different from a template distribution (arrows), i.e. gray areas in the KS-graphs in the middle. P-vaules of the test is plotted below. (C) Estimates of eSIT and eRT versus position of the template distribution relative to the spike from (B) (broken lines represent mean values eSIT = 12.1 ms, eRT = 8.7 ms). (D) The number of traces in template distribution decreases with window length (from same data as in (B) and (C)).

We first considered the V_m_-statistics prior to spikes. The time of the earliest statistical sign of depolarization prior to action potentials we dub “the effective synaptic integration time” (eSIT). This depolarization may be caused by a rise in excitatory conductance or a fall in inhibitory conductance. For each cell, the eSIT was estimated by comparing a template distribution of V_m_ with distributions of V_m_ as a function of time prior to action potentials, as in figure 1. The comparison was attained using a KS-two-sample test at each point in time (method, Figure 3). The template distribution was chosen at a time, t_template_, well before the spikes (arrows Figures 3A and B). The eSIT did not depend systematically on the choice of t_template_ as long as it was at least 10 ms prior to the spike (Figure 3C) even though the number of samples in the distribution decreased (Figure 3D).

### Memory of a spike–eRT

The same statistical test was used to evaluate the impact of a spike by comparing the V_m_-distribution after action potentials with template distributions well before the spike (arrow Figure 3B). In the graph of the KS-test, the gray area represents rejection of the null hypothesis that the V_m_-distribution before and after the spike was statistically similar to the template distribution. When the V_m_-distribution after the spike was indistinguishable from the template distribution, we considered the impact of a spike to have ceased (graph of P-values, Figures 3A and B). Both eRT and eSIT had no clear dependence on t_template_ earlier than ∼20 ms (Figure 3C). Since the number of spikes in the distribution decreased with t_template_ (Figure 3D) the eRT estimate should not be performed with a high t_template_. We chose to calculate the mean eRT over the range 20 ms<t_template_<40 ms.

### Impact of τ_eff_ and AHP on recovery time

Right after the occurrence of an action potential V_m_ is hyperpolarized. The time it takes for V_m_ to re-polarize back to the level prior to the spike depends on the AHP and on the passive effective time-constant of the membrane, which we refer to as τ_eff_. In our attempt to sort out which part of the re-polarization is due to the passive decay of V_m_ and which is due to the cessation of AHP conductance, it is important to estimate τ_eff_ during network activity. The increase in total conductance will also diminish the relative importance of the AHP conductance and the AHP will appear shorter. To quantify the vestige of the AHP under different levels of input conductance we define the *effective After-Hyperpolarisation Period* (eAHP). This period represents the time it takes until the AHP ceases to affect the V_m_ trajectory. If there is no overlap between the passive membrane decay and the eAHP, the eRT is just the sum of τ_eff_ and eAHP, while if there is overlap eRT will be less than the sum:
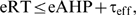



It is important to emphasize the eRT also represents the upper bound on both eAHP and τ_eff_, i.e. eRT≥eAHP and eRT≥τ_eff_. Hence, it was important to determine τ_eff_ for each cell in order to determine the contribution of AHP to the integration process.

### Estimating τ_eff_ during activity

**Figure 4:**
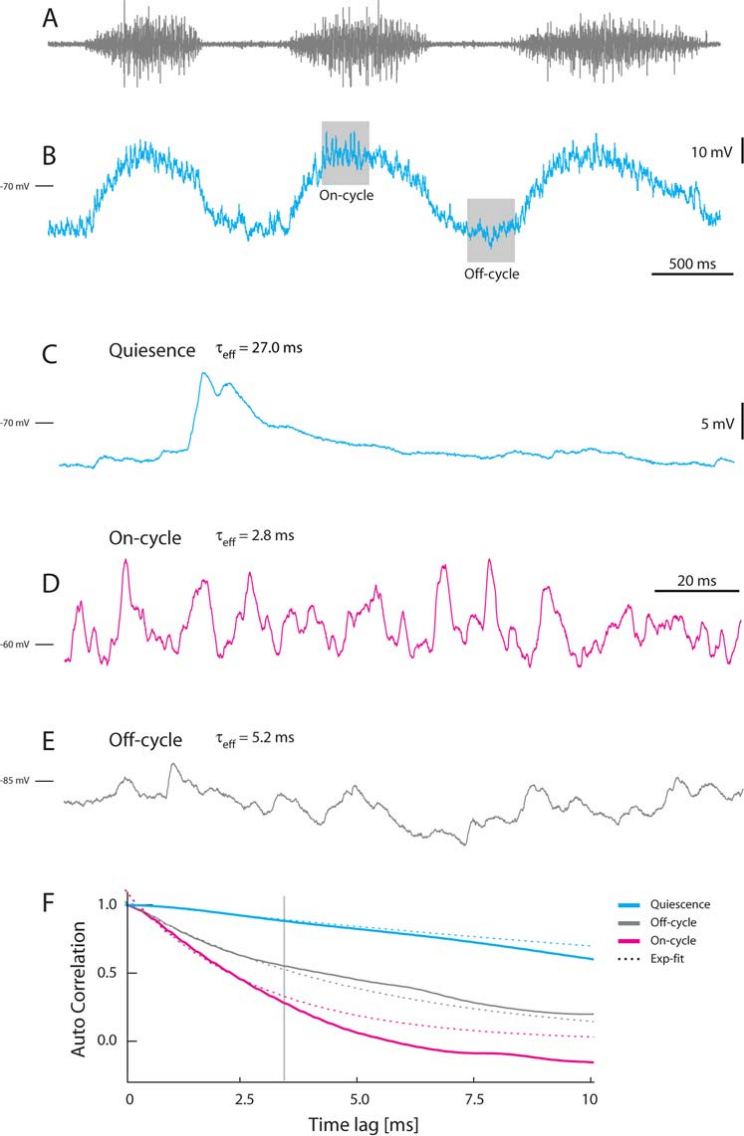
Temporal characteristics of sub-threshold V_m_-fluctuation in motoneurons during quiescence and in the on- and off-cycle during scratching. (A) Hip-flexor nerve recording during scratch. (B) Concurrent V_m_ in MN, spikes avoided with −2.5 nA hyperpolarizing current. Shaded regions mark the selected area of on- and off-cycle illustration below. Sample trace of V_m_ in quiescence (C) (note time-course of spontaneous synaptic potentials) and in the on-cycle (D) and off-cycle (E). D and E are from the shaded boxes in B. (F) The auto-correlation sequence of each sample trace. Blue is from quiescent trace (C), gray is from the off-cycle trace (E), and red is the on-cycle trace (D). The effective time constant of each trace is obtained by fitting an exponential decay function (broken lines) to the initial 3 ms (until the vertical gray line). The time constants are τ_eff_ = 2.8 ms (on-cycle activity), τ_eff_ = 5.2 ms (off-cycle activity) and τ_eff_ = 27.0 ms (quiescence). C–E are on the same time scale.

For the spinal motor activity we differentiated three situations: The quiescent state with little or no synaptic input; the on-cycle with motor nerve activity and spike activity in motoneurons; and the off-cycle, which is at the low point in between the on-cycles (Figure 4A–C). As expected the effective membrane time constant decreased significantly during scratching. For representative data, τ_eff_ was 27 ms in the quiescent state, while it was only 2.8 ms in the On-cycle and 5.2 ms in the off-cycle (Figure 4C–E). Membrane time constants during quiescence are ordinarily estimated from V_m_-decay times after injected current pulses (figure S2). During the dynamic and intense synaptic input in the on-cycle and off-cycle, this method was both difficult and imprecise. Instead, if V_m_ is assumed to follow a stochastic process known as Ornstein-Uhlenbeck-process (OU-process), then maximum likelihood estimation would be the proper way to obtain τ_eff_ [26], [27]. The estimated values of τ_eff_ with this technique are listed in Data and Methods 1 in Data and Methods S1 and see Figures S1 [Supplementary-material pone.0003218.s003]–S3. It turned out that V_m_ did not obey an OU-process (Figure S4) and these estimates of τ_eff_ were systematically much higher than eRT and eSIT. Therefore, τ_eff_ was instead estimated empirically by fitting an exponential decay to the initial part of the auto-correlation sequence (Figure 4F). It was necessary to fit to the initial part (from 0 to 3 ms, vertical gray line) to assure that the auto-correlation lag was small compared to the total length of the sample (200 ms), since the premise of exponential decay is infinite-length of data trace (see Data and Method S1).

### Temporal features for the population

**Figure 5:**
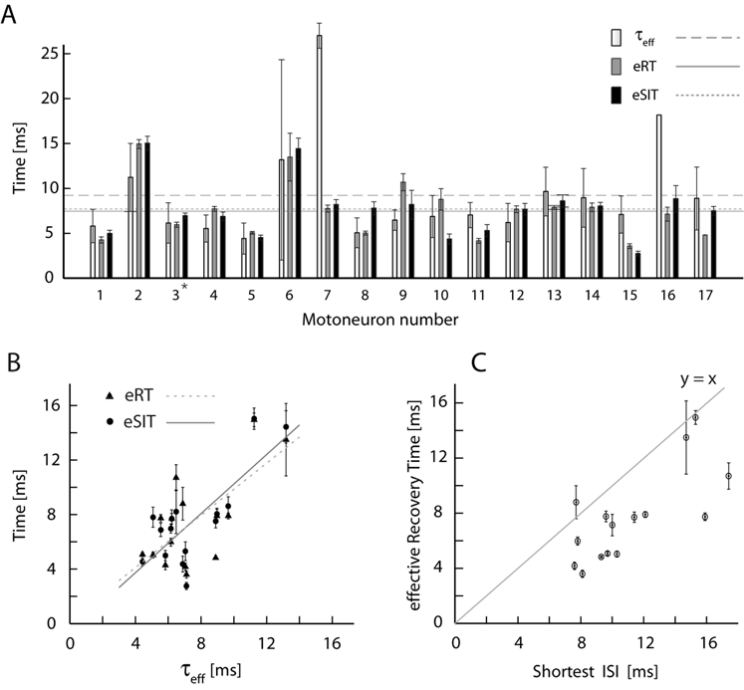
Time constants across the population of MNs. (A) The average τ_eff_ (for ON-cycle), eRT and eSIT (mean±SE) as triplets bars for each cell. Horizontal lines represent population averages, μ = 9.5 ms for τ_eff_, μ = 7.5 ms for eRT, and μ = 7.7 ms for eSIT. Cell 3 is marked with a ★ and the sample cell used in figures 3, 4, 5. (B) eRT and eSIT plotted against τ_eff_ show significant correlation. The lines are linear least square fits. (C) The shortest ISI at zero current injection plotted against the average eRT for each MN. Gray line is where x = y.

The effective synaptic integration times, the effective recovery times and the effective membrane time constants across the population of neurons are listed in Figure 5A. The average eSIT was *μ* = 7.7±0.8 ms and the average eRT was *μ* = 7.5±0.7 ms (mean±SE, n = 17). The median of eSIT and eRT were both 7.7 ms. The eRT was assumed to be approximately equal to the sum of τ_eff_ and the effective AHP (see above). However, the population average of τ_eff_ was *μ* = 9.3±1.4 ms (mean±SE, n = 17) while the median of τ_eff_ was 7.0 ms. In some cases (41%, n = 7/17) τ_eff_ was longer than the corresponding eRT, which suggests two things. First, the reset potentials were closer to the steady state mean V_m_ than the natural fluctuations of V_m_ around the mean, so the decay from reset back to the mean was faster than τ_eff_. Secondly, the eAHP was close to zero or no more than a couple of milliseconds. This value of eAHP is a dramatic decrease from the 200 ms AHP duration previously reported during quiescence [4], [24]. Further, not only did the population spread in eRT across cells correlate with the population spread in eSIT (R^2^ = 0.81), the eRT and the eSIT both had significant correlation with τ_eff_ (Figure 5B) (R_eSIT_^2^ = 0.60, p = 0.0007, R_eRT_^2^ = 0.47, p = 0.005, when ignoring the two outliers, cell 7 and 16). Since eRT is dependent on both τ_eff_ and eAHP, while eSIT is only dependent on τ_eff_, these strong correlations also indicate that the contribution of eAHP to eRT must be minor in most cells.

Hence, the population spread in eRT, eSIT, and τ_eff_ probably reflected different levels of synaptic intensity in different cells. Since the eRT is the major contribution to the refractory period, we expected most of the inter-spike intervals (ISI) to be longer than or equal to the eRT. If this is the case, the data points in a plot of the shortest ISI versus eRT in each cell should fall at the 45°-line or below. Indeed, all points were within their error bars or below (Figure 5C). In this way, the inverse of the eRT represents an upper bound for the spike frequency during network activity.

### Absence of spike frequency adaptation

**Figure 6:**
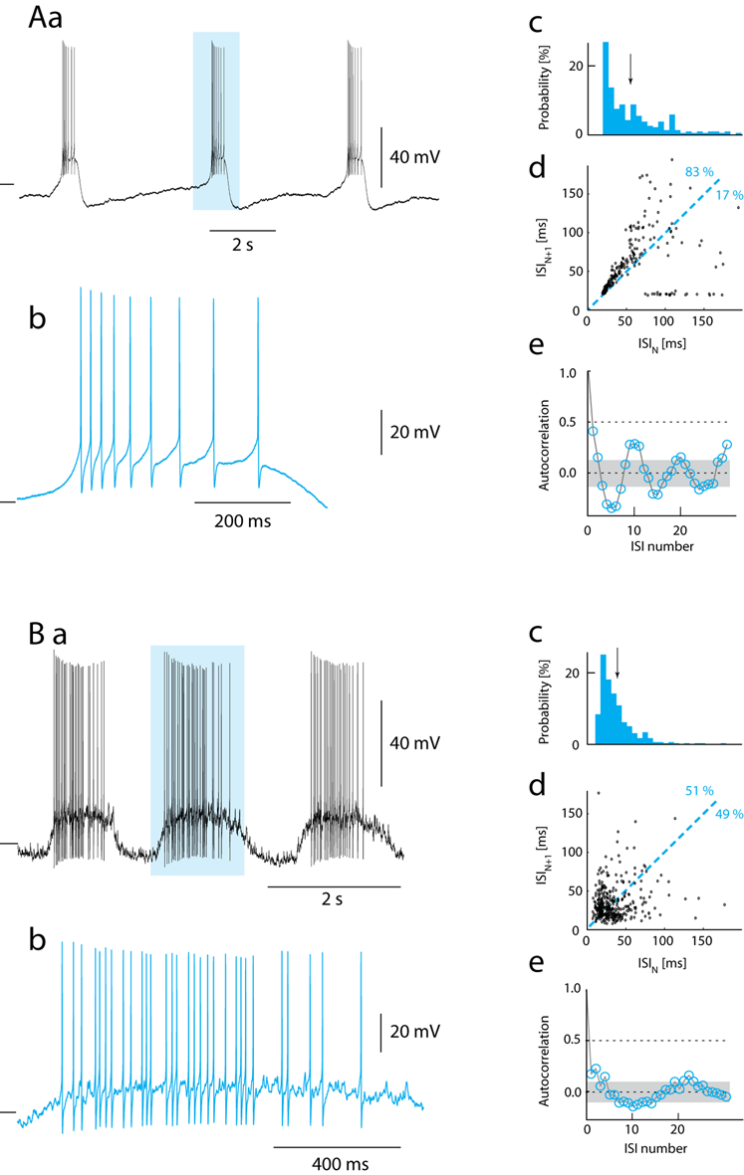
Presence and absence of spike frequency adaptation (SFA) during bursting. (Aa) SFA in intracellular recording from motoneuron in slice during NMDA induced bursting. (Ab) Single burst highlighted in (Aa) show gradual increase in ISI. (Ac) Histogram of ISI. The mean ISI is 55 ms (arrow). (Ad) Plot of ISI_N_ against ISI_N+1_ shows significantly greater proportion of points above than below the ISI_N_ = ISI_N+1_ line (83.2% above, total N = 239), which is evidence of spike frequency adaptation. In addition, ISIs are correlated with their neighbors (correlation coefficient = 0.41), as expected when ISI are influenced by AHP conductance and the burst pattern is reproduced after 10 spikes (Ae). Gray area represents the 5% confidence limit 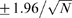 (Ba) Recording from a MN in a functional spinal network during rhythmic motor activity. (Bb) A hightligted cycle from (Ba) shows irregular spike times and no SFA. (Bc) Histogram of ISI. The mean ISI is 34 ms (arrow) (Bd) A plot of ISI_N_ against ISI_N+1_ illustrates no discrepancy of points above and below the ISI_N_ = ISI_N+1_ line (51.3% above, total N = 362), which demonstrates absence of SFA. Furthermore, there is only a marginal correlation of ISI with neighbors (correlation coefficient = 0.18), as expected with negligible AHP conductance (Be). Gray area represents the 5% confidence limit 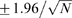. Tick marks to the left represent −80 mV (A) and −60 mV (B). Inter-burst-intervals are omitted in analysis.

The AHP contributes to spike frequency adaptation (SFA) in motoneurons at rest [4], [24]. A classical example of SFA in absence of synaptic input is during bursting induced by N-Methyl-D-Aspartic acid (NMDA). We therefore performed a heuristic comparison between NMDA-bursting in MNs in slices with the bursting induced by synaptic input in functional networks (cf. Figures 6A and 6B). During each burst in the case of NMDA-bursting (Figure 6Aa), spike frequency peaked at onset and adapted to a lower level at the end of the burst (Figure 6Ab). The histogram of interspike-intervals confirmed similarity in firing rate distribution between NMDA-bursting and network bursting [28] (cf. Figure 6Ac and 6Bc). ISI_N_ plotted against ISI_N+1_ displayed a ring-like pattern (Figure 6Ad), as expected from adapting spike trains in each successive burst [29]. Note that the vast majority of points (83%/17%, SE = 6%) are above the 45°-line, which is a consequence of SFA, presumably in large part produced by the prominent AHP. Under these conditions neighboring ISIs were significantly correlated (R^2^ = 0.17) as expected from AHP-mediated adaptation (Figure 6Ae).

**Figure 7:**
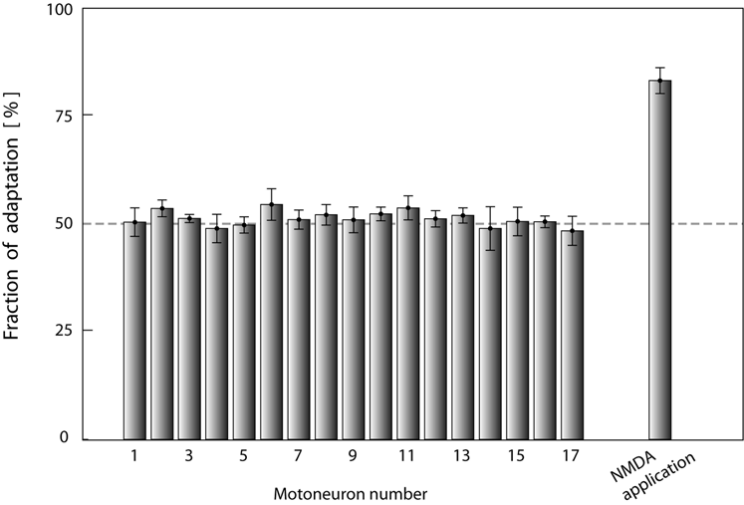
Spike frequency adaptation during NMDA induced bursts but absent during scratching. The number of points above the ISI_N_ = ISI_N+1_ –line in a ISI-return map (Figure. 7Ad and 7Bd) divided by the total number of intervals (×100%) for all MNs including the NMDA induced bursting data from slice experiment for comparison. The network induced bursting have little or no SFA since there is an even amount of points above as below. In contrast, the NMDA-activated bursting has a much larger fraction above the line reflecting the high degree of SFA. Error bars are 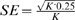, where K total number of ISI.

The firing pattern in MNs during scratching was qualitatively different (Figure 6Ba). Though the distribution of ISI times resembled the NMDA-bursting (cf. Figure 6Bc and 6Ac), spike timing was irregular (Figure 6Bb) and points scattered nearly symmetrically around the 45°-line in the return map (51%/49%, SE = 6%) (Figure 6Bd). This shows that *spike frequency acceleration* was as prevalent as *spike frequency adaptation* during bursts [29] and therefore that the AHP and other intrinsic mechanisms for adaptation or acceleration in spike frequency did not have a detectable influence on the firing pattern during network activity. Furthermore, the correlation between ISI_N_ and ISI_N+1_ was marginal (R^2^ = 0.03) (Figure 6Be), which shows that spikes were driven by a stochastic process rather than by deterministic intrinsic properties. In all 17 motoneurons SFA was insignificant during depolarizing waves, i.e. the numbers above the line were not significantly higher than 50% (Figure 7). Thus, we conclude that the mechanisms causing SFA such as AHP accumulation were not pervasive enough to overcome the increase in synaptic conductance during motor activity.

## Discussion

Spike timing, the principal output of neurons, is determined by interacting synaptic and intrinsic ionic conductances. Recent decades have provided a wealth of information about the intrinsic response properties and their proposed roles in specific cell types in many parts of the nervous system [5], [30], [31]. Properties of the AHP in particular have been linked to classification of fast and slow spiking neurons with clear functional connotations [5], [32]. The present study reports up to ten-fold decrease in the membrane time constant (Figure 4) and severe reduction in the AHP during functional activity (Figure 3 and 5) which emphasize that assertions about the functional significance of intrinsic response properties must be validated in neurons embedded in an active network. In our experimental paradigm, the role of the AHP in spike timing is essentially eliminated and replaced by rapid temporal integration in the high conductance state provided by intense synaptic input (Figures 3, 5, 6). We therefore propose that motor systems perform rapidly changing movements by letting intense synaptic input transiently supplant slow intrinsic properties when necessary.

### Coincidence detection

This transition has previously been referred to as a transition from temporal integration to coincidence detection in sensory perception [33]. Konig and colleagues defined a coincidence detector as a neuron in which the integration time for synaptic potentials is short compared with the average ISI, and a temporal integrator if the reverse is true. Adopting this definition, the motoneurons in the present study were all coincidence detectors during motor activity, since their eSIT and eRT on average are an order of magnitude shorter than their average ISI (cf. Figure 5A and 6Bc, and see Figure S5). The functional effect and benefit of MNs working as coincidence detectors could be to minimize aberrant firing due to the background barrage of noisy synaptic input [28]. The potentially undesirable consequence of having such a coincidence detection scheme is irregular spike patterns, which we indeed did observe (Figure 6). However, irregular patterns are not likely to be a problem, since muscle fibers are slow integrators, and therefore the exact temporal structure of the MN firing, whether irregular and yet fast coincidence detectors or slow regular temporal integrators, is unimportant for securing a smooth contraction.

### Origins of spike pattern

The network mechanisms underlying the phasic spike activity in motoneurons during rhythmic motor behaviors are unknown. It has been hypothesized that certain intrinsic properties are crucial mediators of bursting rhythms in spinal networks [3], [5], [34]. It is also widely accepted that the after-hyperpolarization in MN secures repetitive firing at low rates appropriate for regulation of muscle contraction [3], [4]. The high conductance state, however, compromises the ability to fire repetitively during steady depolarization [11]. This is probably a direct consequence of the reduced slow AHP since the same effect is observed when the AHP is reduced by blocking the underlying ionic current pharmacologically rather than by shunting [35]. Nevertheless, MNs still fired in a broad range of rates in the high conductance state while driven by rapid fluctuations in membrane potential during motor activity (Figure 6B) [13]. Further evidence for fluctuation driven irregular firing is the fact that the shortest ISIs in the active network are longer than the eRT (and thus eAHP) (Figure 6C). This is in contrast to the regular firing and frequency adaptation in motoneurons in slices during NMDA induced bursting (Figure 6A). The reduced AHP amplitude, increased spike time variability and the absence of adaptation during locomotor network activity have also been observed in MNs in the decerebrate cat [36]. In the light of our findings it will be interesting to investigate if the underlying mechanism is modulation of intrinsic properties [1], [24], [37] or parallel increase in excitatory and inhibitory synaptic activity [13], [38].

In conclusion, the rhythm-generation could have two origins: a pattern generating subset of neurons elsewhere in the network in which intrinsic response properties are protected from shunting by intense synaptic bombardment or alternatively, the motor rhythm can be an emergent distributed network phenomenon as suggested for respiratory rhythms [39], [40], i.e. the network bursting hypothesis. Decisive experimental tests of these hypotheses are still missing.

### Caveats of constant current protocols

The role of AHPs in repetitive firing in MNs induced by depolarizing current through the recording electrode has been thoroughly investigated [4], [5], [24]. Based on the linear summation of synaptic potentials under certain experimental conditions [41], [42] this approach takes injected current as a simplifying representation of synaptic input. However, important aspects of synaptic input are overlooked in this approach. First, synaptic variability adds fluctuations as a second moment to V_m_ [22]. Synaptic V_m_-fluctuations may have important computational roles [43]–[45]. Secondly, the effect of surge in conductance from synaptic input is unaccounted for in the constant current protocol (Figure 1). Therefore the functional role of the AHP and other intrinsic response properties should be assessed either in functional networks with real synaptic input, as in the present study, or by dynamic clamp analysis in neurons at rest [7], [8], [46] if a valid estimate of the temporal structure of the functional synaptic conductances is available.

### Balanced state in the spinal cord

Though anatomical evidence suggests an approximate balance between inhibitory and excitatory contacts in cat motoneurons [4], the circuit substrate for variations in the balance of inhibition and excitation during motor activity and its prevalence in other spinal networks is yet unresolved. High-conductance states produced by parallel increase in inhibitory and excitatory synaptic activity is a common occurrence in other functional networks [6], [38], [47]–[51]. In the neocortex, the link between excitation and inhibition is provided by feed-forward and recurrent pathways [48], [49], [52]–[55]. In the spinal cord, recurrent inhibition is unlikely to contribute significantly to the high conductance state in MNs since recurrent collaterals are scarce in the turtle [56], [57] and Renshaw inhibition has not been documented. Feed-forward inhibition provided by Ia inhibitory interneurons can produce synaptic conductance that clearly reduces intrinsic response properties in cat MNs *in vivo* [58]. However, reciprocal inhibition contributes insignificantly to the high conductance state in MNs during breathing [38] and scratching [13]. We propose that inhibitory input to MN is mostly due to local feedforward inhibitory connections, but it remains to be seen if balanced spinal motor networks resemble architectures of the much better investigated networks in other parts of the nervous system [6], [38], [48], [51], [55].

## Materials and Methods

All the experiments were performed in an integrated spinal cord-carapace preparation from the adult turtle except the heuristic control experiment of NMDA-induced spike frequency adaptation in figure 6A, which was performed in a transverse slice from adult turtle. In the integrated preparation the spinal cord remains in the spinal canal with the tactile sensory nerves from the carapace intact. The motor nerves are carefully transected to avoid muscle movements and dissected out to for electro-neurogram recordings. The scratch reflex was activated by mechanical somato-sensory stimulation of selected regions on the carapace (see below) as described [11], [20].

### Integrated preparation

Red-eared turtles (*Trachemys scripta elegans*) were placed on crushed ice for 2 hrs to ensure hypothermic anesthesia. Animals were killed by decapitation and blood substituted by perfusion with a Ringer solution containing (mM): 120 NaCl; 5 KCl; 15 NaHCO_3_; 2 MgCl_2_; 3 CaCl_2_; and 20 glucose, saturated with 98% O_2_ and 2% CO_2_ to obtain pH 7.6. The carapace containing the D4-D10 spinal cord segments was isolated by transverse cuts and removed from the animals, similar to studies published elsewhere [19], [20]. The surgical procedures complied with Danish legislation and were approved by the controlling body under the Ministry of Justice.

### Slice preparation

One mm thick slices of the turtle spinal cord were placed in a chamber for intracellular recording and submerged in and perfused with oxygenated Ringer solution. The pharmacological agent N-methyl-D-aspartate (NMDA) was added to the ringer medium to induce bursting activity (10 µM).

### Recordings

Intracellular recordings in current-clamp mode were performed with an Axoclamp-2A amplifier (Axon Instruments, Union City, CA). Glass pipettes (part no. 30-0066, Havard Apparatus, UK) were pulled with a electrode puller (model P-87, Sutter instrument co., USA) and filled with a mixture of 0.9 M potassium acetate and 0.1 M KCl. Intracellular recordings were obtained from neurons in segment D10. Recordings were accepted if neurons had a stable membrane potential more negative than −50 mV. Data were sampled at 20 kHz with a 12-bit analog-to-digital converter (Digidata 1200, Axon Instruments, Union City, CA), displayed by means of Axoscope and Clampex software (Axon Instruments, Union City, CA), and stored on a hard disk for later analysis. Hip flexor nerve activity was recorded with a differential amplifier Iso-DAM8 (WPI) using a suction pipette. The bandwidth was 100 Hz–1 kHz.

### Activation of network

Mechanical stimulation was performed with the fire polished tip of a bent glass rod mounted to the membrane of a loudspeaker in the cutaneous region known to elicit “pocket scratch” [12] which results in a broad activation of cells [59]. The duration, frequency, and amplitude of the stimulus were controlled with a function generator (Figure 2A). This tactile stimulus induced the scratch-like network activity, which was monitored by the suction electrode nerve recordings from the Hip-flexor nerve (Figure 2B).

### Model data

The data used to illustrate the difference between synaptic-current and synaptic-conductance based fluctuating inputs (Figure 1) was based on a one-compartment model simulation [22] supplemented with Ca^2+^ conductance and a Ca^2+^-activated K^+^-conductance. The synaptic noise was modeled as white current noise in the current-based regime with the heuristic expression,
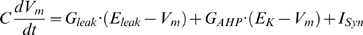
where the fast conductances of the action potential and Ca^2+^-conductance were not shown here for simplicity (for complete description see Data and Methods S1) and the total membrane conductance was
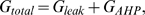
which contains no synaptic component. In this scheme, the synaptic input was represented as a current, I_syn_. The membrane capacitance is C, and G_leak_, E_leak_, G_AHP_, E_K_ are conductance and reversal potential of leak and AHP, respectively.

In a more realistic regime, the high intensity synaptic input was modeled as a conductance [22]. Here, the AHP was reduced as a consequence of the increase in total input conductance (Figure 1). The conductance-based regime applies when the synaptic conductance (G_syn_) is so large that it can no longer be considered small compared with the G_total_ [6]. Heuristically expressed similar to the synaptic current model
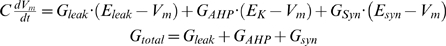
where E_syn_ is the weighted reversal potential of excitatory and inhibitory synaptic reversal potentials and G_syn_ is the sum of both conductances. G_syn_ is competing with G_AHP_ in controlling inter-spike intervals, and if it is large enough it can render G_AHP_ insignificant at steady state:
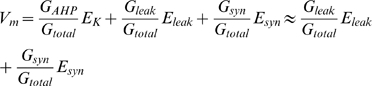
when *G_Syn_*≫*G_AHP_*. The action potentials were largely unaffected by the conductance increase, since fast Na/K conductances of the action potential were much larger. In a simple Hodgkin-Huxley-model added AHP- and Ca^2+^- conductances and including synaptic input as either current noise (I_syn_ = 12 nA, σ_syn_ = 3 nA, OU-simulated with time-constant = 1 ms and D = 0.0005) or conductance noise we verified the theoretical importance of synaptic conductance (Figure 1). The conductance noise was a mixture between inhibition and excitation balanced at −60 mV (see Data and Method S1 for details).

### Evolution of V_m_-distribution

The variable constituting the estimation of effective eSIT and eRT was the membrane potential (V_m_). This variable was stochastic and a sample measurement drawn from an underlying probability distribution function (P_V_), which we assumed had the same statistics in all interspike intervals. The probability distribution depends on time after occurrence of spike and this dependence was a manifestation of intrinsic current generators like SK-channels. Because of the large synaptic fluctuations, it was necessary to look at the distribution P_V_ instead of just isolated instances of V_m_. These fluctuations were assumed uncorrelated from trial to trial, so we could estimate P_v_ by superimposing spikes.

The key assumption is, if the distribution at some given point in time, P_v_(t_1_), is different from the distribution at a later point in time, P_v_(t_2_), then there has been a change in the intrinsic current generation (cf. Figure 3A and B). We selected P_v_ at one point in time (t = t_template_) as a template distribution, which all distributions P_V_(t≠t_template_) were compared with. The P_V_(t_template_) was chosen more than 10 ms before the spike, since this region constitute a background V_m_, and was compared via the Kolmogorov-Smirnov-2 sample test (KS-test) [60] expressed formally as:
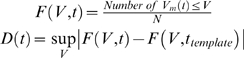



F(V, t) is the empirical cumulative probability distribution function of V_m_. N is the total number of traces (and spikes) used to estimate the distribution at time t (Figure 3D). The maximal difference between the cumulatives, D(t), was the measure for rejecting or accepting the null hypothesis. If the difference was larger than a critical value, then we rejected the null hypothesis that V_m_(t) and V_m_(t_template_) were drawn from the same distribution. The binary test outcome was plotted (Figure 3A and B) where 1 represented rejection (gray area) and 0 represented no rejection of null hypothesis, at a 5% confidence limit. The p-value of the test was plotted below.

### Effective Recovery time

The first point in time after the spike, where the KS-test was zero (i. e. no rejection of hypothesis of same distribution) was where we defined the AHP conductance and other transient intrinsic current generators no longer had a significant impact on the V_m_ and the passive diffusive spread had reach steady state. We dubbed this period *effective recovery time* (*eRT*, arrow in Figure 3B) in analogy to the effective membrane resistance and effective membrane time constant [14], [16], [21], [22]. This recovery time told us how long time after the spike had occurred that there was still a *memory* of the spike in V_m_ (Figure 3B).

### Effective synaptic integration time

Similar to eRT, we could ask how long time prior to the spike, that P_v_ was different from the template distribution. This point represented a net depolarization caused either by reduced inhibition or increased excitation. We named this period *effective synaptic integration time* (eSIT) indicating the time prior to the spike where its occurrence can be predicted (arrow in Figure 3B).

### Location of template

Obviously, the choice of template distribution is important. The template is always chosen prior to the spike. The earlier before the spike we choose the template, the more independent it is. However, there is a trade off, since the inter-spike interval has to be longer than the window between the template distribution and the spike. As a result, the larger the window is, the fewer spikes in a finite dataset will participate in the distribution (Figure 3D). Fortunately, the estimation of both eRT and eSIT is largely independent on the window size (Figure 3C). We chose to average the values of eRT and eSIT from templates 20 to 40 ms prior the spike, since these locations gave nearly constant values (Figure 3C).

### Critique of method

The above described statistical testing of the evolution of P_v_ only accounts for changes that are locked to the occurrence of a single action potential, such as the AHP. Accumulative events that build up over several spikes as e.g. spike frequency adaptation or plateau potentials are not easily accounted for using this statistics. One way to test for slow changes would be to divide the spikes according into several different groups depending on their position in the epoch. These groups could then be compared to evaluate if the distributions have changed. However, we decided this was outside the scope of the present study and it was not necessary since the test of spike frequency adaptation (Figure 6) came out negative.

### Inter-spike interval analysis

The inter-spike intervals were extracted from the intracellular recording during scratch episodes and processed. The auto-correlations were calculated as the normalized covariance function [26], [61] between ISI_N_ and ISI_N+1_, ISI_N+2_, ISI_N+3_ etc (Figure 6d). The test for spike frequency adaptation (Figure 6e) was performed assuming statistical independence of observation of (ISI_N_, ISI_N+1_)-pairs [29] and thus a binomial distribution with chance of 50% above and 50% below the line ISI_N_ = ISI_N+1_. If the number of points above was within a standard deviation of
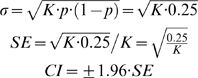
of the 50%-line, as expected from an even binomial distribution [61], there was no significant spike frequency adaptation. K is the total number of points and p is the probability of a point being above the line, when assuming no correlation.

### Data processing

All analysis was performed in Matlab (version 7.3, Mathworks). The data was converted from Axoclamp format to matlab and the spikes were identified and superimposed (Figure 3) to study the V_m_ statistics before and after the spike as described above. KS-testing of the V_m_ distributions was done with the matlab procedure “kstest2.m”. The custom made procedures for calculating eRT and eSIT has been uploaded to mathworks code sharing web site (http://www.mathworks.com/matlabcentral/) with the name “eRT.m” for the interested reader. The remaining matlab code is available on request.

## Supporting Information

Data and Method S1 (0.07 MB DOC)

Figure S1 (10.67 MB TIF)

Figure S2 (4.21 MB TIF)

Figure S3 (10.05 MB TIF)

Figure S4 (9.28 MB TIF)

Figure S5 (3.40 MB TIF)

## References

[1] Miles GB, Hartley R, Todd AJ, Brownstone RM (2007). Spinal cholinergic interneurons regulate the excitability of motoneurons during locomtion.. PNAS.

[2] Rekling JC, Funk GD, Bayliss DA, Dong XW, Feldman JL (2000). Synaptic control of motoneuronal excitability.. Physiological reviews.

[3] Grillner S (2006). Biological pattern generation: The cellular and computational logic of networks in motion.. Neuron.

[4] Kernell D (2006). The motoneurone and its muscle fibres.

[5] Toledo-Rodriguez M, El Manira A, Wallen P, Svirskis G, Hounsgaard J (2005). Cellular signalling properties in microcircuits.. TINS.

[6] Destexhe A, Rudolph M, Pare D (2003). The high-conductance state of neocortical neurons in vivo.. Nature Rev Neurosci.

[7] Wolfart J, Debay D, Masson GL, Destexhe A, Bal T (2005). Synaptic background activity controls spike transfer from thalamus to cortex.. Nature Neuroscience.

[8] Fernandez FR, White JA (2008). Artificial Synaptic Conductances Reduce Subthreshold Oscillations and Periodic Firing in Stellate Cells of the Entorhinal Cortex.. J Neurosci.

[9] Borg-Graham LJ, Monier C, Fregnac Y (1996). Voltage-clamp measurement of visually-evoked conductances with whole-cell patch recordings in primary visual cortex.. J Physiology (Paris).

[10] Borg-Graham LJ, Monier C, Y. F (1998). Visual input evokes transient and strong shunting inhibition in visual cortical neurons.. Nature.

[11] Alaburda A, Russo R, MacAulay N, Hounsgaard J (2005). “Periodic high-conductance states in spinal neurons during scratch-like network activity in adult turtles”.. J Neurosci.

[12] Robertson GA, Stein PSG (1988). Synaptic control of hindlimb motoneurones during three forms of fictive scratch reflex in the turtle.. J Physiol.

[13] Berg RW, Alaburda A, Hounsgaard J (2007). Balanced inhibition and excitation drive spike activity in spinal half-centers.. Science.

[14] Barret JN (1975). “Motoneuron dendrites: role in synaptic integration”.. Federation proceedings.

[15] Destexhe A, Rudolph M, Fellous J-M, Sejnowski TJ (2001). Fluctuating synaptic conductances recreate in vivo-like activity in neocortical neurons.. Neuroscience.

[16] Bernander OJDR, Martin KAC, Koch C (1991). Synaptic background activity influences spatiotemporal integration in single pyramidal cells.. PNAS.

[17] Shefchyk SJ, Jordan LM (1985). Motoneuron input-resistance changes during fictive locomotion produced by stimulation of the mesencephalic locomotor region.. J Neurophysiol.

[18] Perreault MC (2002). Motoneurons have different membrane resistance during fictive scratching and weight support.. J Neurosci.

[19] Keifer J, Stein PSG (1983). Invitro motor program for the rostral scratch reflex generated by the turtle spinal-cord.. Brain research.

[20] Alaburda AJH (2003). Metabotropic modulation of motoneurons by scratch-like spinal network activity.. J Neurosci.

[21] Rapp M, Yarom Y, Segev I (1992). The impact of parallel fiber background activity on the cable properties of cerebellar purkinje cells.. Neural Computation.

[22] Tiesinga PHE, Jose JV, Sejnowski TJ (2000). Comparison of current-driven and conductance-driven neocortical model neurons with Hodgkin-Huxley voltage-gated channels.. Physical review E.

[23] Destexhe A, Pare D (1999). Impact of network activity on the integrative properties of neocortical pyramidal neurons in vivo.. J Neurophysiol.

[24] Brownstone RM (2006). Beginning of the end: repetitive firing properties in the final common pathway.. Progress in Neurobiology.

[25] Guillamon A, McLaughlin DW, Rinzel D (2006). Estimation of synaptic conductances.. J Physiology Paris.

[26] Tuckwell HC (1988).

[27] Ditlevsen S, Lansky P (2005). Estimation of the input parameters in the Ornstein-Uhlenbeck neuronal model.. Physical review E.

[28] Mazzoni A, Broccard FD, Garcia-Perez E, Bonifazi P, Ruaro ME (2007). On the dynamics of the spontaneous activity in neuronal networks.. PLoS one.

[29] Rodieck RW, Kiang NY-S, Gerstein GL (1962). Some quantitative methods for the study of spontaneous activity of single neurons.. Biophysical J.

[30] Llinás RR (1988). The intrinsic electrophysiological properties of mammalian neurons: insights into central nervous system function.. Science.

[31] Häusser M, Spruston N, Stuart GJ (2000). Diversity and dynamics of dendritic signaling.. Science.

[32] Bartos M, Vida I, Jonas P (2007). “Synaptic mechanisms of synchronized gamma oscillations in inhibitory interneuron networks”.. Nat Rev Neurosci.

[33] Konig P, Engel AK, Singer W (1996). Integrator or coincidence detector? The role of the cortical neuron revisited.. TINS.

[34] Delgado-Lezama R, Hounsgaard J (1999). Adapting motoneurons for motor behavior.. Progress in Brain Research.

[35] Hounsgaard J, Kiehn O, Mintz I (1988). Response properties of motoneurones in a slice preparation of the turtle spinal cord.. J Physiol.

[36] Brownstone RM, Jordan LM, Kriellaars DJ, Noga BR, Shefchyk SJ (1992). On the regulation of repetitive firing in lumbar motoneurones during fictive locomotion in the cat.. Exp Brain Res.

[37] Schmidt BJ (1994). Afterhyperpolarization modulation in lumbar motoneurons during locomotor-like rhythmic activity in the neonatal rat spinal cord in vitro.. Exp Brain Res.

[38] Parkis MA, Dong X-W, Feldman JL, Funk GD (1999). Concurrent inhibition and excitation of phrenic motoneurons during inspiration: Phase-specific control of excitation.. J Neuroscience.

[39] Del Negro CA, Morgado-Valle C, Feldman JL (2002). Respiratory rhythm: an emergent network property?. Neuron.

[40] Feldman JL, Del Negro CA (2006). Looking for inspiration: New perspectives on repiratory rhythm”.. Nat Rev Neurosci.

[41] Granit R, Kernell D, Lamarre Y (1966). Algebraic summation in synaptic activation of motoneurones firing in the “primary range” to injected currents.. J Physiol.

[42] Powers RK, Binder MD (2000). Summation of effective synaptic currents and firing rate modulation in cat spinal motoneurons.. J Neurophysiol.

[43] Chance FS, Abbott LF, Reyes AD (2002). Gain modulation from background synaptic input.. Neuron.

[44] Shadlen MN, Newsome WT (1998). The variable discharge of cortical neurons: Implications for connectivity, computation, and information coding.. J Neurosci.

[45] Stein RB, Gossen ER, Jones KE (2005). Neuronal variability: Noise or part of the signal?. Nat Rev Neurosci.

[46] Prinz AA, Abbott LF, Marder E (2004). The dynamical clamp comes of age.. TINS.

[47] Csicsvari J, Hirase H, Czurko A, Mamiya A, Buzsaki G (1999). Oscillatory coupling of hippocampal pyramidal cells and interneurons in the behaving rat.. J Neurosci.

[48] Haider B, Duque A, Hasenstaub AR, McCormick DA (2006). Neocortical network activity in vivo is generated through a dynamical balance of excitation and inhibition.. J Neurosci.

[49] Wehr M, Zador AM (2003). Balanced inhibition underlies tuning and sharpens spike timing in auditory.. Nature.

[50] Baca SM, Marin-Burgin A, Wagenaar DA, Kristan WB (2008). Widespread inhibition proportional to excitation controls the gain of a leech behavioral circuit.. Neuron.

[51] Monier C, Fournier J, Fregnac Y (2008). In vitro and in vivo measures of evoked excitatory and inhibitory conductance dynamics in sensory cortices.. J Neurosci Meth.

[52] Kapfer C, Glickfeld LL, Atallah BV, Scanziani M (2007). Supralinear increase of recurrent inhibition during sparse activity in the somatosensory cortex.. Nature Neuroscience.

[53] Silberberg G, Markram H (2007). Disynaptic inhibition between neocortical pyramidal cells mediated by Martinotti cells.. Neuron.

[54] Monier C, Chavane F, Baudot P, Graham LJ, Fregnac Y (2003). Orientation and direction selectivity of synaptic inputs in visual cortical neurons: a diversity of combinations produces spike tuning.. Neuron.

[55] Marino J, Schummers J, Lyon DC, Schwabe L, Beck O (2005). Invariant computations in local cortical networks with balanced excitation and inhibition.. Nature Neuroscience.

[56] Ruigrok TJ, Crowe A, Donkelaar HJ (1984). Morphology of lumbar motoneurons innervating hindlimb muscles in the turtle Pseudemys scripta elegans: an intracellular horseradish peroxidase study.. J Comp Neurol.

[57] Chmykhova NM, Adanina VO, Karamian OA, Kozhanov VM, Vesselkin NP (2005). Comparative study of spinal motoneuron axon collaterals.. Brain Research Bulletin.

[58] Hyngstrom AS, Johnson MD, Miller JF, Heckman CJ (2007). Intrinsic electrical properties of spinal motoneurons vary with joint angle.. Nature Neuroscience.

[59] Berkowitz A (2001). “Broadly tuned spinal neurons for each form of fictive scratching in spinal turtles”.. J Neurophysiol.

[60] Press WH, Teukolsky SA, Vetterling WT, Flannery BP (1992). Numerical recipes in fortran-The art of scientific computing. 2nd edition.

[61] Taylor JR (1982). An introduction to error analysis-The study of uncertainties in physical measurements.

