# Supplementary material for: Intense Synaptic Activity Enhances Temporal Resolution in Spinal Motoneurons
Source: PLoS One. 2008 Sep 16;3(9):e3218. doi: 10.1371/journal.pone.0003218 (PMC2528963; doi:10.1371/journal.pone.0003218)
Supplement: Data and Method S1 — (0.07 MB DOC) [file pone.0003218.s001.doc]

Supporting Data and methods for the article:

**Intense synaptic activity enhances temporal resolution in spinal motoneurons**

Rune W. Berg^1^, Susanne Ditlevsen^2^ and Jorn Hounsgaard^1^

^1^Department of Neuroscience and Pharmacology, University of Copenhagen, Blegdamsvej 3, DK-2200, Copenhagen N, DENMARK

^2^Department of Mathematical Sciences, University of Copenhagen, Universitetsparken 5, DK-2100, Copenhagen Ø, DENMARK

**Data and Methods 1.**

**Tau from MLE during ON-cycle.** We estimated the membrane time-constants using the Maximum Likelihood estimation (MLE) procedure in addition to using the auto-correlation relaxation fitting (figure 4F). The results for both procedures during ON-cycles are plotted together for comparison in Figure S1. The horizontal lines are the population mean for the MLE and the autocorrelation exponential fit, respectively. The MLE values are roughly 1 order of magnitude larger than the autocorrelation fitting values. The mean value of the autocorrelation fit was 9.3 ± 1.4 ms (mean ± standard error) and the median was 7.0 ms.

**Tau during quiescence.** For comparison we calculated the time-constants without synaptic inputs. Current pulses were injected into the cells and the corresponding V_m_ deflections were analyzed by fitting an exponential decay with constant off-set. The time-constant of this fit was the passive time-constant during quiescence and they are plotted in Figure S2 with a mean of 17.5 ± 1.7 ms (mean ± standard error) across the population and a median of 16.5 ms.

**Tau during OFF-cycle.** For comparison we calculated the time-constants via the autocorrelation fits for the OFF-cycles across a population. The values are plotted in supplementary figure 3. The sample mean was 11.2 ± 1.0 ms (mean ± standard error) and the median was 11.3 ms.

**Spectral features of fluctuations**

The OU-process, the most commonly assumed and applied stochastic process to describe the synaptic V_m_ fluctuations, is a smoothed version of Steins model where inhibitory and excitatory synaptic input is modeled as Poisson processes, and the membrane potential decays towards a resting potential in absence of input (a leaky term). The estimated values of τ_eff_ with this technique are listed above in Figure S1. It turned out that V_m_ in motoneurons were poorly described by an OU-process. The roll-off slopes in the power spectrum of the sample data were about -3.2 during on-cycle (Figure S4B) and -2.8 during off-cycle (Figure S4C), which was substantially higher than the anticipated -2 (Figure S4D). Notice the high frequency peak in power, which was common, but not always present. The roll-off slopes for the entire population of MN clustered around -3 for on-cycle and -2.3 for off-cycle (Figure S4E). We attribute the difference in slope to the dendritic arbor of MNs which deviates from the one-compartment assumption and causes a steeper power spectrum. Further, the deviation from the OU-process was also reflected in the fact that estimates of τ_eff_ were an order of magnitude higher than the empirical eRT. Therefore, τ_eff_ was instead estimated by fitting an exponential decay to the initial part of the auto-correlation sequence (main manuscript Figure 4F).

**Inter-spike intervals.** We measured the mean inter-spike intervals for each cell, while ignoring the inter-burst intervals. The values are shown in Figure S5 with the sample standard deviation. The average value is approximately an order of magnitude higher than the average time constants (Figure S5A).

**Data Methods 2.**

**Estimating V_m_ time-constants for NON-OU-processes.** An OU-process has a power spectrum with a roll-off slope of -2 on the log-log scale (Figure S4). Our data had a steeper roll-off slope of about -3, which indicates that the one-dimensional process probably is a too simple description of these data and therefore gave poor estimates of the time-constant. A good indicator of the temporal process is either the location of the roll-off in the power-spectrum, or the decay of the auto-correlation function. For slow processes, the auto-correlation has a long decay time and vice versa for fast processes. Thus, we used the initial segment of the empirical autocorrelation function and fitted an exponential decay. The autocorrelation estimate is only reliable up to some lag value << the number of observations in the sample (which is 250 ms at 10 khz = 2500). The recommended maximum lag value differ among authors, but 20-40 lags are generally accepted as long as N_observations_ > 160. Here, we used 30 lags of the sampling interval of 0.1 ms corresponding to 3 ms of the autocorrelation to fit the mono-exponential fit (Figure 4F). Another guideline for estimating this critical lag time is the general formula used in the computer languages R and S-plus:

τ_lag_ ≤ 10 log_10_(N_observations_)*Δ

where N_observations_ is the number of samples that the the autocorrelation estimate is based on, and Δ is again the sampling interval (R Development Core Team, 2007). This also suggest use up to 10*Log(250 ms/.1 ms/sample)*0.1 ms =3.3 ms ≈ 3 ms of the autocorrelation to fit the mono-exponential fit (Figure 4F).

**Maximum likelihood estimation of effective time contant.**

Membrane time constants during quiescence can be estimated from V_m_-decay times in response to injected current pulses. In the dynamic situation of intense synaptic input this method is less expedient. Instead, we use maximum likelihood estimation of the effective membrane time constant by assuming V_m_ is governed by an Ornstein-Uhlenbeck process. The subthreshold membrane dynamics during massive synaptic input (synaptic fluctuations) can be approximately characterized by the Langevin equation i.e. by a passive membrane potential equation with a stochastic input, representing random synaptic input. This is referred to as an Ornstein-Uhlenbeck (OU) process. The Fokker-Planck equation describes the evolution of the probability distribution of V_m_, which is Gaussian. The transition probability turns out to be particularly useful for our analysis, in which each action potential acts as a reset of the V_m_ distribution, and our task is to follow the evolution of the distribution from this point. If the decay of P_V_ back to a steady state takes longer after the action potential, than expected from an OU process, the extra time delay must be due to spike after-effects. The power spectra were calculated for windowed data and their slopes were found by linear fitting in the roll-off region (30-500 hz).

**Data Methods 3**

**Details of the model neuron.** The complete principal model equation of figure 1 including the fast conductances was

**Where we set C=1*10^-15^ F, G_leak_ = 0.3 μS, E_leak_ = -54 mV. During the conductance noise the total conductance was 8 μS.**

**Na^+^ current. The Na^+^-current was modeled as I_Na_ = G_Na,peak_*m_Na_^3^*h_Na_*(V_m_–E_Na_), with activation parameter m_Na_, from dm_Na_/dt = (m_Na-inf_ – m_Na_)/τ_mNa_, where τ_mNa_ = 1/[α_m_+β_m_], α_m_ = (V_m_+40)/[10*(1-exp(-(V_m_+40)/10)], β_m_ = 4*exp(-0.0556*(V_m_+65)), m_Na-inf_ = α_m_/(α_m_ + β_m_ ), and with inactivation parameter, dh_Na_/dt = (h_Na-inf_ – h_Na_)/τ_hNa_, τ_hNa_ = 1/[α_h_ + β_h_], α_h_ = 0.07*exp(-0.05*(V_m_+65)), β_h_ = 1/{1+exp(-0.1*(V_m_+35))} , h_Na-inf_ = α_h_/(α_h_ + β_h_). G_Na,peak_ = 480 nS and E_Na_ = 50 mV.**

**K-current. The K-current was modeled as I_K_ = G_K,peak_*m_K_^4^*(V_m_ – E_K_), with activation parameter m_K_, from dm_K_/dt = (m_K-inf_–m_K_)/τ_mK_, where τ_mK_ = 1/[α_mK_+β_mK_], α_mK_ = (V_m_+55)/[100*(1-exp(-(Vm+55)/10)], β_m_ = 0.125*exp(-0.0125*(V_m_+65)), m_K-inf_ = α_mK_/(α_mK_+β_mK_). G_K,peak_ = 100 nS and E_K_= -77 mV.**

**Ca^2+^-current. The Ca^2+^-current was modeled as I_ca_ = G_Ca,peak_*m_Ca_*h_Ca_*(V_m_ - E_Ca_), with activation parameter m_Ca_, from dm_Ca_/dt = (m_Ca-inf_ – m_Ca_)/τ_mCa_ , where τ_mCa_ = 7.8/[exp((V_m_+6)/16) + exp(-(V_m_+6/16))], m_Ca-inf_ = 1/[1+exp(-(V_m_-3)/8)] and inactivation parameter h_Ca_ = 0.01/[0.01 + [Ca^2+^]_in_] where [Ca^2+^]_in_  is the intracellular Ca^2+^-concentration. G_Ca,peak_ = 0.1 nS and E_Ca_ = 141 mV. The Ca^2+^ removal via buffers, pumps and diffusion was modeled as a simple exponential decay, d[Ca^2+^]_in_ /dt = {[****Ca^2+^]_in-inf_ -[Ca^2+^]_in_}/ τ_Ca-removal_, with τ_Ca-removal_ = 10 ms. [Ca^2+^]_in-inf_= -0.005*I_Ca_*τ_Ca-removal_ .**

**AHP-current. The AHP-current is modeled as a non-inactivating Ca^2+^-dependent K^+^-current, I_AHP_ = G_AHP,peak_*m_AHP_*(V_m_ – E_K_), where dm_AHP_/dt = (m_AHP-inf_ – m_AHP_)/τ_AHP_, τ_AHP_ = 30 000/{ [Ca^2+^]_in_^2^*1.25*10^8^+25 000}, m_Ca-inf_ =[Ca^2+^]_in_^2^*1.25*10^8^/{ [Ca^2+^]_in_^2^*1.25*10^8^+25 000}. G_AHP,peak_= 7.99 nS. These parameters were adapted values published elsewhere (Koch 1999, Gerstner and Kistler 2002).**

**Synaptic conductance**

**The synaptic conductances in the high intensity scheme were modeled as Ornstein-Uhlenbeck processes with the stochastic differential equation in compact notation written as**

**and integrated via exact numerical integration (Gillespie 1996) where G is either the inhibitory or excitatory conductance G_i_ and G_e_, and respective relaxation time constants τ_i_= 2 ms, τ_e_= 1 ms. The values used for both were σ =0.0005 nS^2^/ms and the mean conductances were balanced so V_m_ = -68 mV and G_i, mean_ = G_total_ – G_leak_ - G_e, mean_ and G_e, mean_ =[G_tot_*(V_m_-E_i_) +G_leak_*(E_i_-E_leak_)]/(E_e_-E_i_), and G_tot_= 8 nS. All computer simulations were performed in Matlab (version 7.3, Mathworks) with integration time step of 0.05 ms.**

**References**

**Ditlevsen S, Lansky P (2005)** "Estimation of the input parameters in the ornstein-uhlenbeck neuronal model" Physical review E, 71, 011907

**Gerstner W, Kistler WM (2002)** "Spiking neuron models: Single neurons, populations, plasticity" Cambridge University Press.

**Gillespie DT (1996)** "Exact numerical simulation of the Ornstein-Uhlenbeck process and its integral" Physical Review E 54(2): 2084-2091

**Koch C (1999)** "Biophysics of computation: information processing in single neurons. Oxford: Oxford university press

**R Development Core Team (2007).** R: A language and environment for statistical computing. R Foundation for Statistical Computing, Vienna, Austria. ISBN 3-900051-07-0, URL [http://www.R-project.org](http://www.R-project.org/).

**Tuckwell HC (1988)** "Introduction to theoretical neuroscience, Vol. 2" Cambridge, Cambridge University Press
