# Supplementary figures and images for: Intense Synaptic Activity Enhances Temporal Resolution in Spinal Motoneurons
Source: PLoS One. 2008 Sep 16;3(9):e3218. doi: 10.1371/journal.pone.0003218 (PMC2528963; doi:10.1371/journal.pone.0003218)

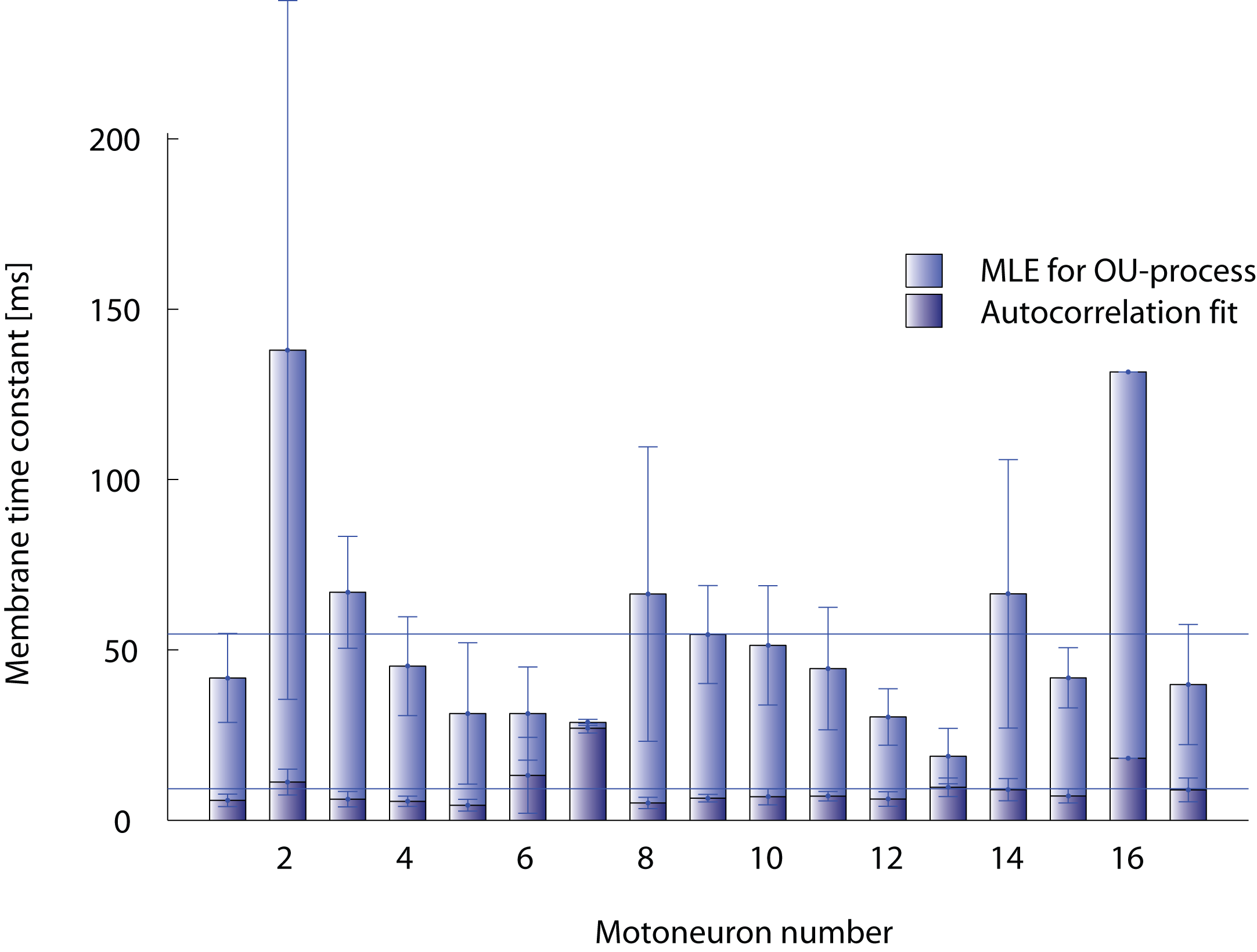

Supplement: Figure S1 — (10.67 MB TIF) [file pone.0003218.s002.tif]

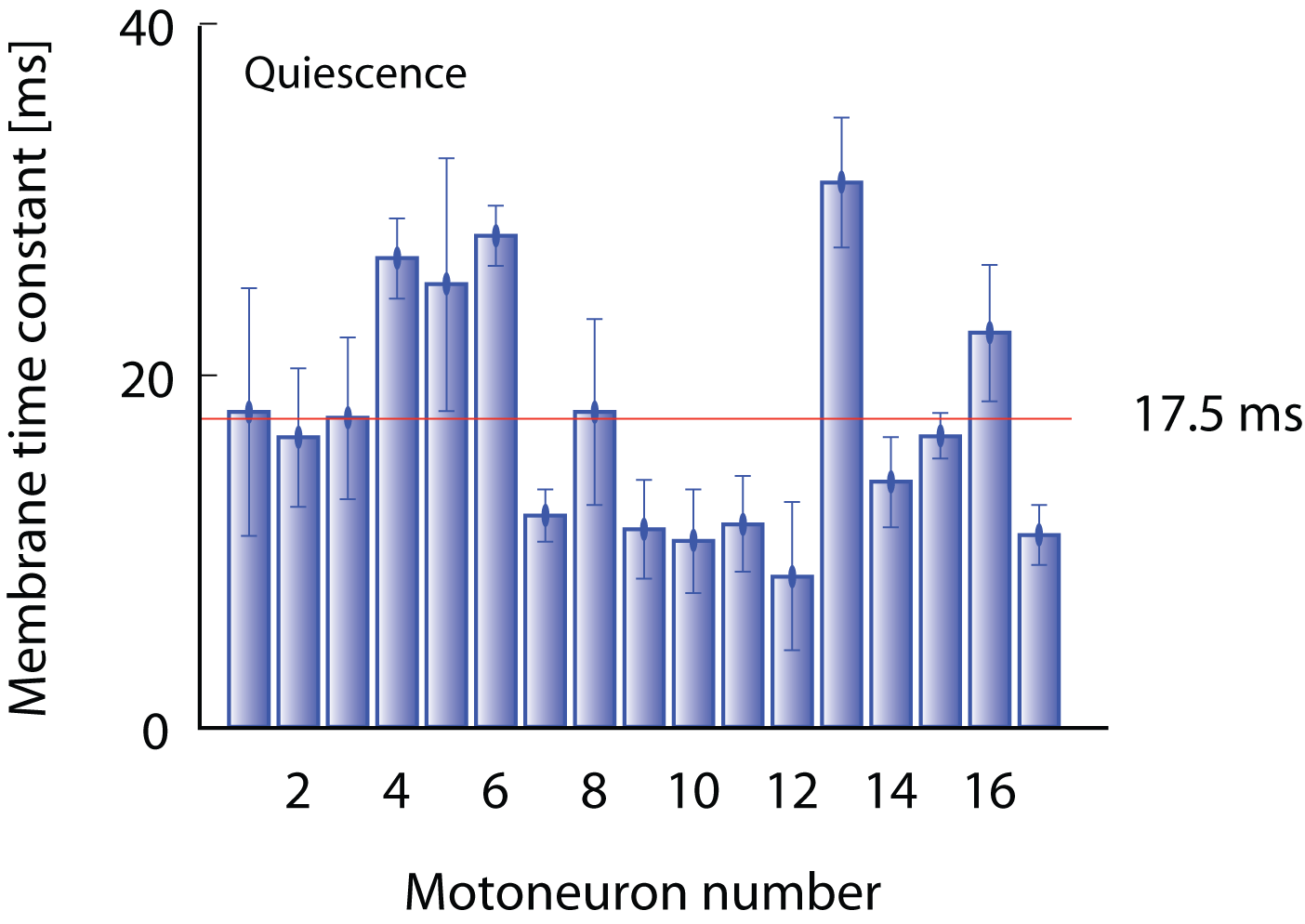

Supplement: Figure S2 — (4.21 MB TIF) [file pone.0003218.s003.tif]

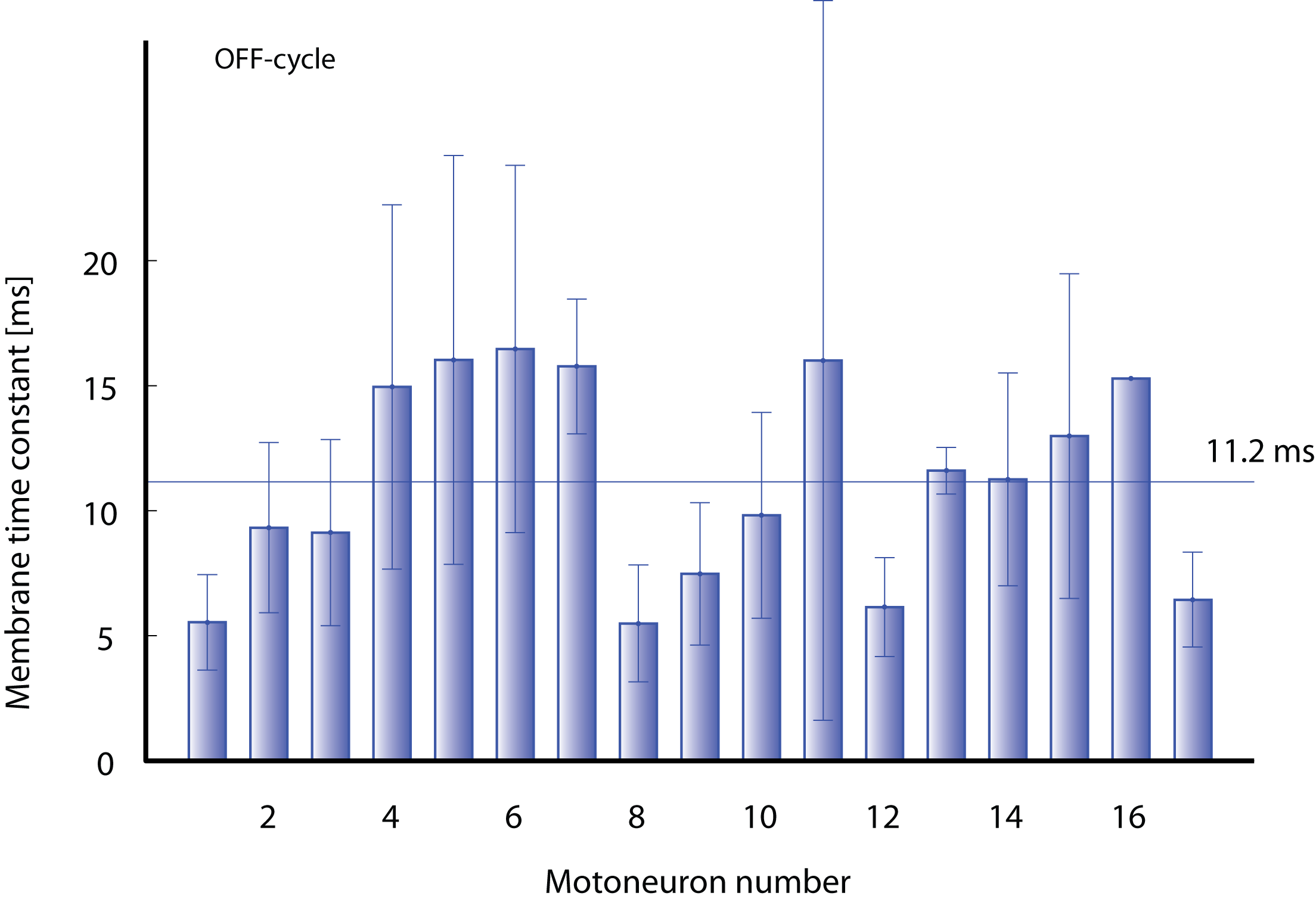

Supplement: Figure S3 — (10.05 MB TIF) [file pone.0003218.s004.tif]

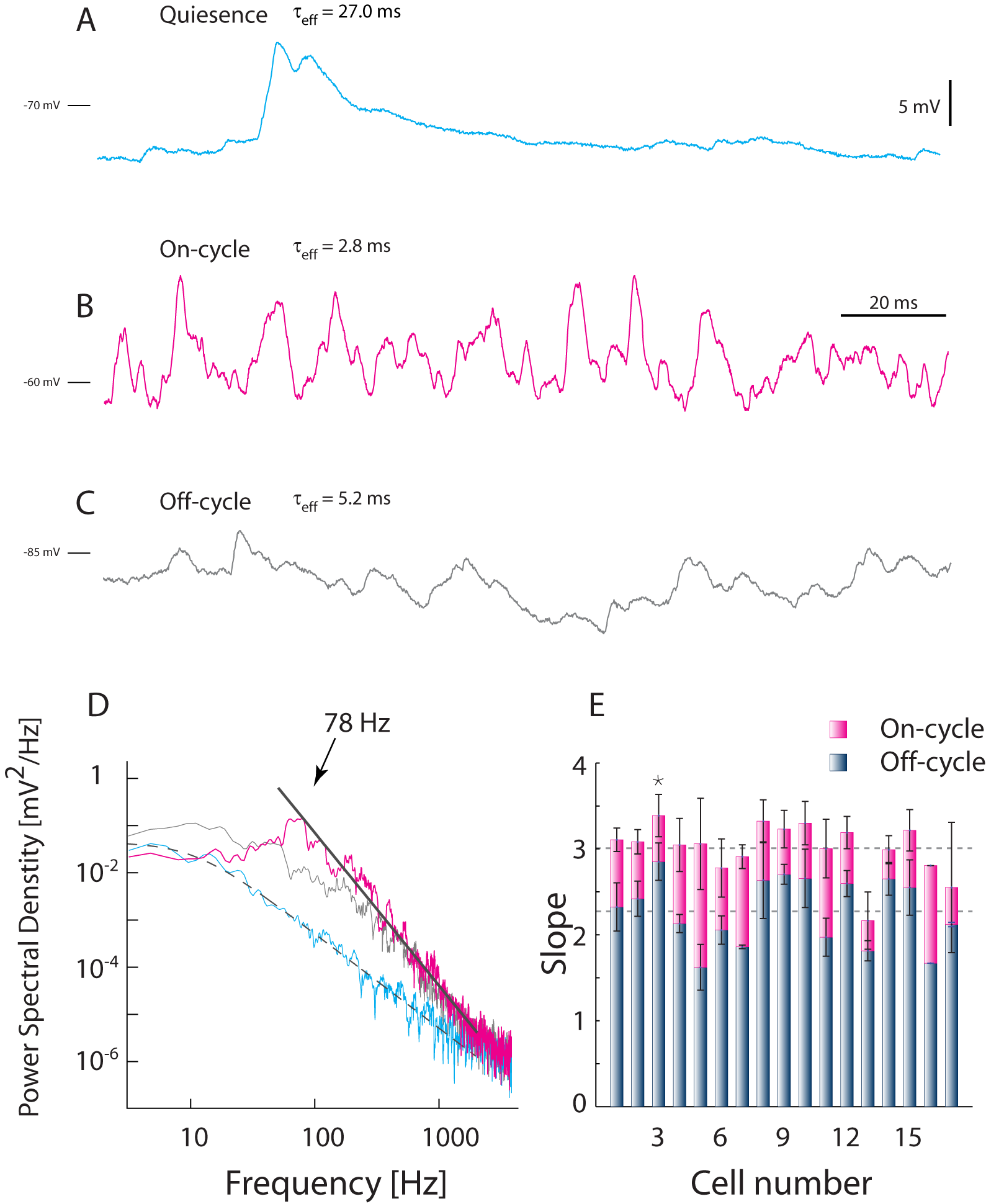

Supplement: Figure S4 — (9.28 MB TIF) [file pone.0003218.s005.tif]

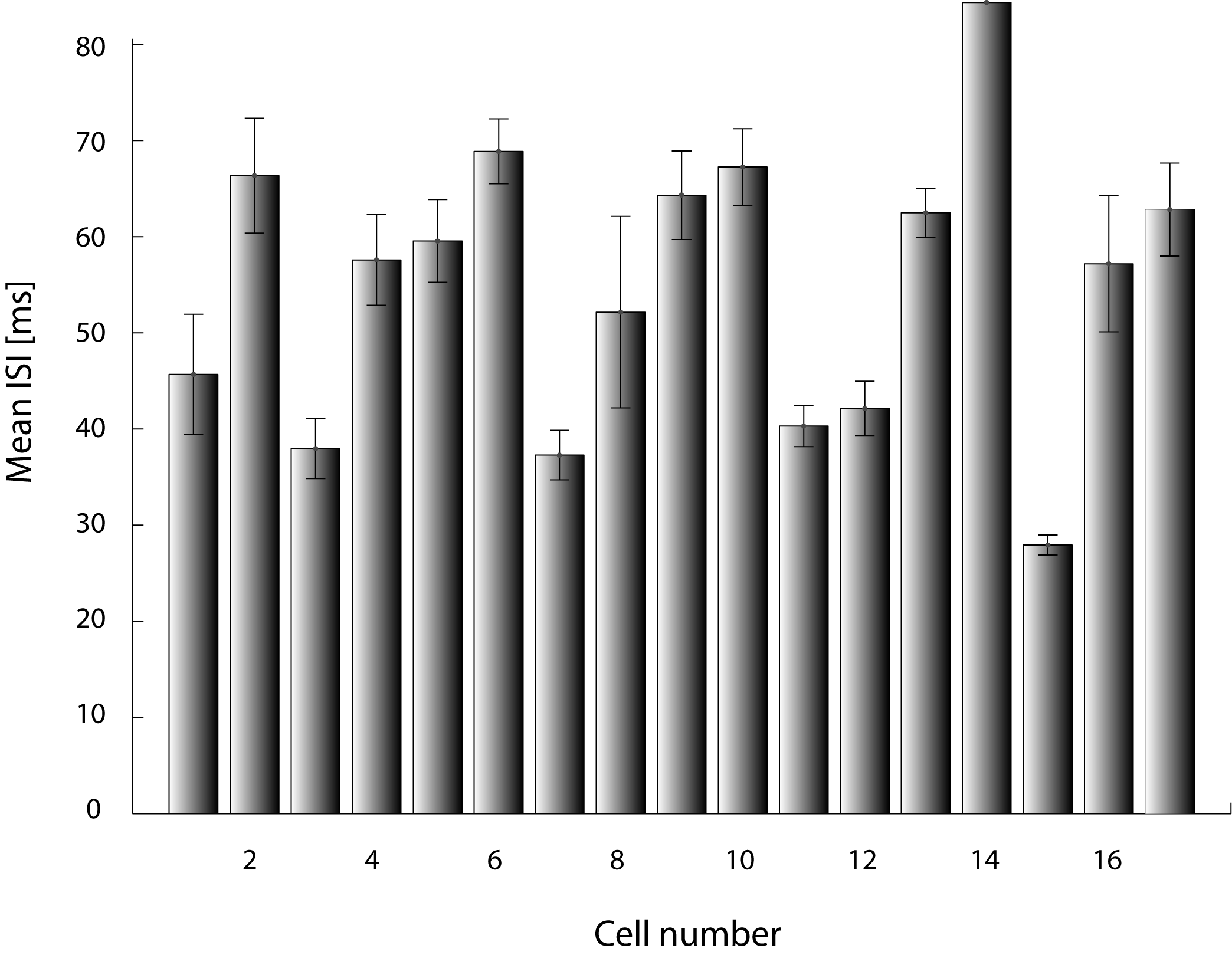

Supplement: Figure S5 — (3.40 MB TIF) [file pone.0003218.s006.tif]
